# Evaluation of survival in patients after pancreatic head resection for ductal adenocarcinoma

**DOI:** 10.1186/1471-2482-13-12

**Published:** 2013-04-22

**Authors:** Marius Distler, Felix Rückert, Maximilian Hunger, Stephan Kersting, Christian Pilarsky, Hans-Detlev Saeger, Robert Grützmann

**Affiliations:** 1 Department of General, Thoracic and Vascular Surgery, University Hospital Carl Gustav Carus, Technical University Dresden, Fetscherstrasse 74, Dresden 01307, Germany; 2 Surgical Department, University Hospital Mannheim, Heidelberg University, Mannheim, Germany

**Keywords:** Pancreatic cancer, Tumor marker, Surgery, Whipple procedure, Pylorus-preserving pancreatoduodenectomy (PPPD)

## Abstract

**Background:**

Surgery remains the only curative option for the treatment of pancreatic adenocarcinoma (PDAC). The goal of this study was to investigate the clinical outcome and prognostic factors in patients after resection for ductal adenocarcinoma of the pancreatic head.

**Methods:**

The data from 195 patients who underwent pancreatic head resection for PDAC between 1993 and 2011 in our center were retrospectively analyzed. The prognostic factors for survival after operation were evaluated using multivariate analysis.

**Results:**

The head resection surgeries included 69.7% pylorus-preserving pancreatoduodenectomies (PPPD) and 30.3% standard Kausch-Whipple pancreatoduodenectomies (Whipple). The overall mortality after pancreatoduodenectomy (PD) was 4.1%, and the overall morbidity was 42%. The actuarial 3- and 5-year survival rates were 31.5% (95% CI, 25.04%-39.6%) and 11.86% (95% CI, 7.38%-19.0%), respectively. Univariate analyses demonstrated that elevated CEA (p = 0.002) and elevated CA 19–9 (p = 0.026) levels, tumor grade (p = 0.001) and hard texture of the pancreatic gland (p = 0.017) were significant predictors of a poor survival. However, only CEA >3 ng/ml (p < 0.005) and tumor grade 3 (p = 0.027) were validated as significant predictors of survival in multivariate analysis.

**Conclusions:**

Our results suggest that tumor marker levels and tumor grade are significant predictors of poor survival for patients with pancreatic head cancer. Furthermore, hard texture of the pancreatic gland appears to be associated with poor survival.

## Background

The prognosis for patients with cancer of the pancreatic head remains poor. Currently, tumor resection is the only therapeutic option to achieve long-term survival.

However, only a small number of patients (30-40%) present a resectable tumor at the time of diagnosis. The overall 5-year survival after pancreatic head resection for cancer ranges between 10 and 25% [1-3]. Adjuvant chemotherapy, which improves patient survival, is routinely used [4,5]. The following characteristics have been reported to be significant prognostic factors for patient survival after tumor resection: age, tumor size, nodal and margin status and tumor grade [2,6-8]. Pancreatic surgery, specifically pancreatoduodenectomy (PD), has been identified to be a ‘formidable’ operation in earlier years [9]. The operation can be performed safely, and postoperative mortality in some specialized pancreatic centers is currently less than 5% [10,11].

Reduced mortality was achieved by concentrating pancreatic surgeries in specialized centers because pancreatic surgery is technically demanding and places high demands on the perioperative management [12,13]. Another important factor for reducing mortality and morbidity is better patient selection. Clinical decision-making will be increasingly influenced by evidence-based medicine [14].

The 5-year survival rate can be significantly improved for patients with pancreatic cancer when surgery is possible. However, some patients relapse shortly after the resection and exhibit a limited life span even after R0 resection.

To better assess the risks and benefits of surgical treatment, it is necessary to analyze the factors that might influence or determine which patients have limited survival.

The present study reports the short- and long-term outcome of 195 consecutive pancreatic head resections due to pancreatic cancer from a single German pancreatic center. Univariate and multivariate analyses were performed to examine the factors affecting survival.

## Methods

### Patients

Eight fellowship-trained pancreatobiliary surgeons performed 672 consecutive PDs between October 1993 and November 2008 in our department; the period of observation was 1993 to 2011. We excluded patients who underwent palliative bypass or pancreatic resections for pancreatic cancer in the body and tail of the pancreas, distal cholangiocarcinoma, duodenal carcinoma, neuroendocrine tumors, cyst-adenocarcinoma, solid and papillary tumors, and metastatic tumors. The final pathological diagnosis confirmed ductal pancreatic adenocarcinoma (PDAC) in 195 (29%) of the remaining patients. The demographic characteristics are summarized in Table 1.

**Table 1:** Patient cohort demographic and clinical data (n = 195)

	**n=**
**Sex** (m/f)	103 (53%)/92 (47%)
**Age y** (±SD)	67.0 (± 9.7) 95% CI 63.3-66.1
**Hypertension** (yes/no)	100 (51.3%)/95 (48.7%)
**Preoperative diabetes mellitus**	
(yes/no)	69 (35.4%)/126 (64.6%)
**Obstructive jaundice** (yes/no)	159 (81.5%)/36 (18.5%)
**Preoperative biliary stent** (yes/no)	139 (71.3%)/56 (28.7%)
**Alcohol abuse** (yes/no)	64 (32.8%)/131 (67.2%)
**Nicotine abuse** (yes/no)	35 (17.9%)/160 (82.1%)
**Pack-years** (±SD)	20 (± 6.5 y)
**BMI** (m/kg^2^)	24.8 (± 3.6) 95% CI 24.5-25.5
**General condition**	
Good	150 (76.9%)
Mediocre	43 (22.1%)
Poor	2 (1.0%)

### Operations

The head resection surgeries analyzed in the study included 69.7% pylorus-preserving pancreatoduodenectomies (PPPD) and 30.3% standard Kausch-Whipple pancreatoduodenectomies (Whipple). The decision for one of the approaches (either Whipple or PPPD) was made during the operation. The primary goal of every operation was en bloc R0 tumor resection. In all the patients, a lymphadenectomy was performed along the hepatoduodenal ligament, common hepatic artery, vena cava, interaortocaval and right side of the superior mesenteric artery. In cases with portal vein involvement, a venous resection was performed to achieve R0-resection. Patients with arterial infiltration by the tumor were stated to be locally irresectable. Thrombosis of the portal vein was always a contraindication for pancreatic head resection. The two-layer invagination technique was used for pancreatic anastomosis in all the cases as previously described [14]. We routinely placed drains intraoperatively. All the patients were staged preoperatively with CT and/or MRI and transabdominal ultrasound, and the PD patients were routinely observed at the Intensive Care Unit (ICU). The drains were removed after exclusion of a postoperative pancreatic fistula (POPF). Postoperative complications were treated symptomatically.

### Data collection

The medical records from a prospective database of patients who underwent PDs for PDAC were analyzed retrospectively for each case. In accordance with the guidelines for human subject research, approval was obtained from the Ethics Committee at the Carl Gustav Carus University Hospital. All the operated patients singed inform consent agreements before surgery. The survey data were complemented with the clinical notes of the patients’ physicians and surgeons. Details regarding the deceased patients were obtained from family members or from the general practitioner. The postoperative follow-up time was three years or until the death of the patient.

Patient characteristics and parameters used for statistical analysis are listed in the supplementary information (Additional file 1: Table S1). The postoperative events and clinical outcomes were recorded prospectively and analyzed retrospectively. The tumor-stage designation was categorized according to the TNM system of the Union Internationale Contre le Cancer (UICC 2007).

### Definitions

Perioperative mortality was defined as in-hospital mortality. Postoperative pancreatic hemorrhage (PPH) was categorized according to the ISGPS consensus definition. [15]. Delayed gastric emptying (DGE) was classified according to the definition suggested by the ISGPS [16]. Postoperative pancreatic fistula (POPF) was defined according to the ISGPF criteria [17].

### Statistical analysis

The statistical analyses were performed using SPSS for Windows, version 15.0 (SPSS, Inc., Chicago, IL). All clinical and pathological characteristics were stratified to build categorical or nominal variables. CEA and CA19-9 were grouped according to the cutoff values used in our center (cut-off levels CEA and CA 19–9: ≤3 ng/ml and ≤75 U/ml). Other variables such as age and BMI were grouped according to previous publications [8]. The thresholds used for categorization were based on previously described thresholds in the literature and/or recursive partitioning as previously described [18]. Continuous data are presented as 95% confidence intervals (95% CI) and standard deviation (SD). The univariate examination of the relationship between the assessed criteria and survival was performed with a χ^2^-test. To assess the impact of the different parameters on survival, we utilized a 3-year survival rate. The estimates of patient survival were generated using the Kaplan-Meier method. The comparisons of survival were performed using the log-rank test. Student’s *t*-tests (ratio scale) and Fisher’s exact tests (ordinal scale) were utilized for comparisons between groups. Ordinal-scaled variables were compared using the chi-square test. Significant factors (at P < 0.10) at the univariate level were entered into the multivariate model. A Cox regression analysis with stepwise backwards elimination based on the likelihood ratios was employed to test for independent predictors of survival. A *p*-value <0.05 was considered significant.

## Results

### Patient demographics and preoperative parameters

From 1993 to 2008, 195 patients underwent pancreatic head resections (PD) due to ductal adenocarcinoma of the pancreas at our institution. The patients were observed from 1993 to 2011. The patient characteristics are described in Table 1. An obstructive jaundice appeared on average 4 weeks before the operation (± 2.3 weeks) in 159 patients (81.5%), and 139 of the patients were preoperatively treated with a biliary stent (71.3%). The maximal bilirubin concentration was 17.26 mg/dl (± 15.9 mg/dl). Weight loss was observed in 119 (61.0%) patients, and the average preoperative weight loss was 8.55 kg (± 4.57 kg). The average onset of weight loss was 8.0 weeks before the operation (± 9.8 weeks).

### Intraoperative parameters

In most of the cases, the indication for operation was suspicion of malignancy (98.5%). The median postoperative hospital stay was 19.02 days (range 7–100) and included a median postoperative ICU stay of 5.2 days (range 0–69). In 29.7% of the cases, a partial resection of the portal vein (or superior mesenteric vein) was necessary. The mean duration of the pancreatic resection was 420.88 ± 99.0 minutes (range: 234–874 min) (Table 2).

**Table 2:** Indications for operations and performed procedures (n = 195)

Indication for operation*	
Pain (%)	1.5
Suspicion of malignancy (%)	98.5
Obstructive jaundice (%)	20.0
Gastric outlet obstruction (%)	1.0
***Performed procedures***	
Whipple (%)	30.3
PPPD (%)	69.7
Resection of the SMV/portal vein (%)	29.7
Mean operative time (minutes) SD	420.88 ± 99.0
Postoperative hospital stay (days)	19.02 (range 7–100)
Postoperative ICU stay (days)	5.24 (range 0–69)

^*^Multiple answers were possible.

(*SMV*, Superior mesenteric vein; *PPPD*, Pylorus-preserving pancreaticoduodenectomy; *ICU*, Intensive care unit).

### Morbidity and mortality

The occurrence of perioperative mortality was 4.1% (8 patients) in the 195 patients who underwent resection for pancreatic cancer. During the postoperative course, 81 patients (42%) developed one or more complications. Most of the complications were minor (30%).

Grade B delayed gastric emptying was observed in 13 patients (6.7%), and grade C delayed gastric emptying was observed in 5 patients (2.6%). Ten patients developed grade B (5.1%) POPF, and 3 patients developed grade C POPF (1.5%). Grade B PPH was observed in 5 patients (2.6%), and grade C PPH was observed in three patients (1.5%). Table 3 presents the morbidity and mortality after PD.

**Table 3:** Morbidity/outcome after PD due to PDAC of the pancreatic head

***Complication/morbidity***	Patients with complication^*^
	***n = 81 (42%)***
**POPF**	
Grade B	n = 10 (5.1%)
Grade C	n = 3 (1.5%)
**PPH**	
Grade B	n = 5 (2.6%)
Grade C	n = 3 (1.5%)
**DGE**	
Grade B	n = 13 (6.7%)
Grade C	n = 5 (3.6%)
**Other complications**	
Wound infections	n = 30 (15.4%)
Postoperative pneumonia	n = 10 (5.1%)
Pancreatitis	n = 8 (4.1%)
Cholangitis	n = 3 (1.5%)
Urinary tract infection	n = 7 (3.6%)
**Anastomotic leakage**	
Hepaticojejunostomy	n = 8 (4.1%)
Pancreatojejunostomy	n = 16 (8.2%)
**In-hospital mortality**	n = 8 (4.1%)

^*^Multiple answers were possible.

Forty-eight of the patients developed one complication, 18 patients developed two complications, seven patients developed three complications, and eight patients developed more than three complications.

### Histological analysis of the specimen

The tumor stage was pT1 in 7 (3.6%) patients, pT2 in 12 (6.1%) patients, pT3 in 173 (88.8%) patients and pT4 in 3 (1.5%) patients; the most frequent postoperative UICC 2002 stages were IIa and IIb. In 138 cases (70.8%), an R0 resection was certified by pathohistological examination of the specimen (R1: n = 42 (21.5%); R2: n = 10 (5.1%); Rx: n = 5 (2.6%)). Nodal disease was diagnosed in 129 (66.2%) of the 195 patients, 66 (33.8%) patients were node negative, 124 (63.6%) patients had a pN1 status, and 5 (2.6%) patients had a pN1b status. In 15 patients, an M1 situation was confirmed in the final pathological evaluation. In all the cases, the M1 status was due to interaortocaval lymph nodes (Table 4).

**Table 4:** Pathological features and tumor classification (n = 195)

**TNM (2007)**	
**Tumor stage**	
pT1	7 (3.6%)
pT2	12 (6.1%)
pT3	173 (88.8%)
pT4	3 (1.5%)
**Nodal status**	
pN0	66 (33.8%)
pN1	124 (63.6%)
pN1b	5 (2.6%)
**Interaortocaval metastasis**	
M1	15 (7.7%)
**Resectional status**	
R0	138 (70.8%)
R1	42 (21.5%)
R2	10 (5.1%)
Rx	5 (2.6%)
**Perineural invasion**	
PN0	76 (39.0%)
PN1	116 (59.5%)
PNx	3 (1.5%)
**Tumor differentiation**	
G1	6 (3.1%)
G2	102 (52.3%)
G3	82 (42.0%)
G4	5 (2.6%)

The PDAC was well differentiated (G1) in 6 (3.1%) patients, intermediately differentiated (G2) in 102 (52.3%) patients, poorly differentiated (G3) in 82 (42.0%) patients and undifferentiated (G4) in 5 (2.6%) patients (Table 4).

### Survival

**Figure 1:**
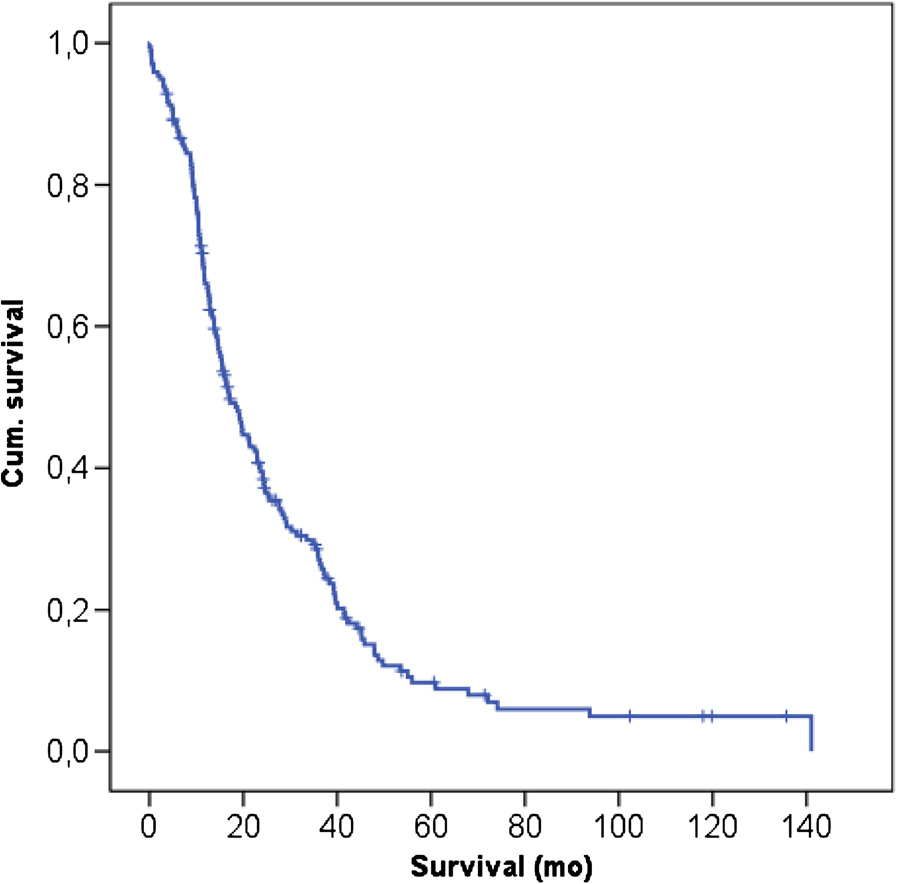
Kaplan-Meier analysis of overall survival of patients with PDAC of the pancreatic head after PD; the 3- and 5-year survival rates were 31.5% (95% CI, 25.04%-39.6%) and 11.86% (95% CI, 7.38%-19.0%), respectively.

To date, 163 of the 195 patients have died; 16 of the patients have died due to other causes and were censored for the survival analysis. The actuarial 3- and 5-year survival rates were 31.5% (95% CI, 25.04%-39.6%) and 11.86% (95% CI, 7.38%-19.0%), respectively. The median overall survival was 17.08 months (95% CI, 14.0%-20.1%) (Figure 1).

Adjuvant therapy was not routinely used in our center until 2003. Seventy-nine (40.5%) of the patients received postoperative adjuvant therapy. The median survival for patients without adjuvant CTx was 16.4 months (95% CI, 11.6- 21.2), and the median survival was 21.0 months (95% CI, 14.2-27.9) for patients with adjuvant CTx, which was not significant (*p* = 0.931).

### Univariate survival analysis

In the univariate analysis, we correlated different parameters with the 3-year survival rate. CEA >3 ng/ml (p = 0.002), CA 19–9 >75 U/ml (p = 0.026) levels, tumor grade 3 (p = 0.001) and hard texture of the pancreatic gland (p = 0.017) were identified as significant predictive factors of poor patient survival. The lymph node ratio, T-stage or R-status (R1/R2 resection) were not found to be significant factors in univariate analysis. Table 5 summarizes the findings of the univariate analysis.

**Table 5:** Univariate analysis of factors that influenced 3-year survival after PD due to PDAC of the pancreatic head

	**3-year survival**	***p *Value**
**Age (years)**		0.073
<60 (n = 74)	n = 22 (30%)	
61-65 (n = 100)	n = 18 (18%)	
>65 (n = 21)	n = 2 (9%)	
***CEA (ng/ml)***		***0.002***
***<4 (n = 151)***	***n = 39 (26%)***	
***>4 (n = 36)***	***n = 1 (3%)***	
***CA 19–9 (U/ml)***		***0.026***
***<24 (n = 39)***	***n = 13 (33%)***	
***>24 (n = 151)***	***n = 26 (17%)***	
**T- stage**		0.894
pT1 n = 7	n = 2 (28%)	
pT2 n = 12	n = 2 (17%)	
pT3 n = 173	n = 37 (21%)	
pT4 n = 3	n = 1 (33%)	
**R-status**		0.155
R0 n = 138	n = 30 (22%)	
R1 n = 42	n = 8 (19%)	
R2 n = 10	n = 1 (10%)	
**Lymph node-ratio**		0.709
<0.2 (n = 134)	n = 30 (22%)	
≥0.2 (n = 60)	n = 12 (20%)	
***Tumor grade***		***0.001***
***G1 (n = 6)***	***n = 4 (67%)***	
***G2 (n = 102)***	***n = 21 (20%)***	
***G3 (n = 82)***	***n = 12 (15%)***	
***G4 (n = 5)***	***n = 2 (40%)***	
***Pancreas texture***		***0.017***
***Hard n = 33***	***n = 2 (6%)***	
***Soft n = 161***	***n = 40 (25%)***	
Preoperative diabetes (n = 69)	n = 12 (17%)	0.365
Nicotine abuse (n = 35)	n = 6 (17%)	0.651
Hypertension (n = 100)	n = 18 (18%)	0.163
BMI >35 (n = 3)	0	0.631
POPF grade B (n = 10)	n = 4 (40%)	0.228
POPF grade C (n = 3)	n = 1 (33%)	0.387

### Multivariate survival analysis

All the factors that were significant in the univariate analyses at the p < 0.10 level (CEA, CA 19–9, age, texture of the pancreas and tumor grade) were tested using multivariate analysis. However, only the CEA level >3 ng/ml (p < 0.001) and tumor grade 3 (p = 0,013) could be identified as independent risk factors for patient survival (HR 2.350 and 1.346, respectively) (Table 6).

**Table 6:** Multivariate analysis of factors that influenced 3-year survival in patients after PD for PDAC of the pancreatic head

		**HR**	**95% Confidence Interval HR**		**P value**
			**Lower**	**Upper**	
**Step 1**	Age	1.205	0,916	1,549	0.162
	CEA >3 ng/ml	2.293	1,488	3,628	<0.001
	CA 19–9 >75 U/ml	1.130	0,721	1,671	0.560
	Tumor-grade 3	1.312	1,032	1,685	0.027
	Pan. Texture (hard)	1.046	0,590	1,713	0.866
**Step 2**	Age	1.203	1,489	3,626	0.164
	CEA >3 ng/ml	2.297	0,916	1,547	<0.001
	CA 19–9 >75 U/ml	1.136	0,724	1,667	0.539
	Tumor-grade 3	1.313	1,032	1,685	0.027
**Step 3**	Age	1.215	0,917	1,547	0.142
	CEA >3 ng/ml	2.258	1,490	3,627	<0.001
	Tumor-grade 3	1.330	1,035	1,683	0.565
**Step 4**					
	CEA >3 ng/ml	2.350	1,518	3,639	<0.001
	Tumor-grade 3	1.346	1,064	1,702	0.013

## Discussion

In the present study, we retrospectively analyzed the long-term survival of patients undergoing PD for carcinoma of the pancreatic head in a single, high-volume center. The aim of this study was to identify predictive factors for long-term survival. The characteristics of the patient cohort were similar to previous reports [12,19]. The head resections included PPPD and Whipple procedures. The most common indication for operation was suspected malignancy, and most of the patients presented obstructive jaundice due to a tumor in the pancreatic head. All the patients exhibited histologically confirmed PDAC in the final examination of the specimen.

We observed a perioperative mortality of 4.1% in our study group, which was within the range of previous reports and indicates that the procedure is safe when performed in a hospital setting [12,19,20]. Surgical complications were observed in 42% of the patients undergoing PD. The high morbidity might have resulted from our comprehensive data acquisition. Our prospective pancreatic database includes surgical and unspecific complications. Most of the analyzed complications exhibited only minor effects on patient health such as wound infection, which was the most common surgical complication, or delayed gastric emptying (DGE). Severe complications (as defined by the ISGPS), such as grade C POPF, PPH or anastomotic leakage, were observed in 10% (n = 19) of the patients (Table 3). The complication rate is similar to previous studies [12,19]. Considering the complication rate, the indication for pancreatic resection should be performed carefully.

During univariate analyses, the elevated tumor marker levels of CEA and CA 19–9, the texture of the pancreas (hard) and the tumor grade (grade 3) were identified as significant factors with negative prognostic effects on patient survival. Multivariate analysis demonstrated that a CEA level > 3 ng/ml (p < 0,005) and tumor grade 3 (p = 0,027) were independent predictive factors for patient survival. CEA and CA19-9 are the most studied serum tumor markers for the diagnosis and prognosis of pancreatic adenocarcinoma [21]. CEA is known to exhibit low sensitivity in screening PDAC [22]. However, other authors hypothesized that high levels might be associated with the existence of occult metastasis or locally advanced diseases in patients with PDAC. Therefore, previously high levels of CEA could be associated with incurability in patients with pancreatic cancer [23,24]. Although CEA might not be appropriate for screening, its serum level should be determined in patients prior to operation. High serum levels of CEA should be considered by the surgeon in cases where respectability or operability is questionable.

Levels of carbohydrate antigen (CA) 19–9, a tumor-associated glycoprotein, are elevated in approximately 85% of patients with PDAC [25], and serum CA19-9 measurements can be used for diagnostic purposes (i.e., as a predictor of resectability or as a marker of recurrent disease after resection) [26]. Both CA19-9 and CEA can be used to predict survival after pancreatic resection [27,28]. In an analysis by Hartwig et al., CA-19-9 levels greater than 400 U/ml were identified as one of the strongest negative survival predictors [29]. However, cholestasis is known to influence serum tumor marker concentrations. Both CA19-9 and CEA undergo biliary excretion, and serum levels may artificially increase due to biliary obstruction caused by cancer masses [30]. Because many patients experienced obstructive jaundice in our study cohort, our results, particularly the CA19-9 results, may be biased. However, in multivariate analysis, CA19-9 could not be identified as a significant predictor of survival in patients with PDAC.

According to our data, tumor differentiation plays a prominent role for survival in pancreatic cancer, as already shown in other tumor entities. Tumor differentiation was an independent prognostic factor in multivariate analysis [1,8,31]. Contrary to these results, we did not observe an influence of the TNM classification or the resectional status, similarly to previous publications [1,8]. It is unclear why these parameters were not correlated with survival. Data published by Esposito et al. suggest that most resections in pancreatic cancer are R1 resections [32]. As a result, other researchers in this field refer to pancreatic cancer as a “systemic disease”. While the classification as a systemic disease may be immoderate for a solid tumor, it shows that aggressive infiltration and metastasizing are important hallmarks of pancreatic cancer. These hallmarks might be more pronounced in tumors with poor differentiation, which might also explain why our results did not identify the lymph node ratio as a significant factor as previously published by Riediger et al. The lymph node ratio may be diagnostically relevant similar to other tumor entities. However, tumor grading appears be a more relevant indicator of the patient’s prognosis as shown in our analysis.

Furthermore, the texture of the pancreas was identified to be a predictor of survival in our analysis. Soft texture of the gland was related to a good prognosis (p < 0.017). This sudden finding is especially difficult to interpret because pancreatojejunal anastomosis is safer in a fibrotic pancreas compared with a soft and friable normal pancreas with a narrow main pancreatic duct [14,33]. In patients with a soft pancreas, chronic pancreatitis is usually not present, and the patients may exhibit better organ function, which results in a better prognosis. Tumors derived from a chronic pancreatitis (associated with hard texture) were more aggressive or exhibited more aggressive pathophysiology than carcinomas arising from a normal pancreas. Because no significance for the texture was shown in the multivariate analysis, the parameter might be a surrogate for another variable influencing outcome. Furthermore, the evaluation of pancreatic texture must be considered, especially in retrospective analyses, because it is very subjective and easy to overestimate.

The actuarial overall 3- and 5-year survival rates were 31.5% (95% CI, 25.04%-39.6%) and 11.86% (95% CI, 7.38%-19.0%), respectively. These findings are similar to those reported in the literature [1,12,29]. There was no statistical significance between the subgroups of patients with or without adjuvant chemotherapy [median survival: without adjuvant CTx 16.4 months (95% CI, 11.6- 21.2 months) vs. with adjuvant CTx 21.0 months (95% CI, 14.2-27.9 months) (p = 0.931)], which may be due to the heterogeneity of the chemotherapeutic regimes. Approximately 50% of our patients received adjuvant chemotherapy (i.e., mainly with gemcitabine). Although long-term survival may be achieved in only a minority of patients, the complete surgical resection of pancreatic adenocarcinoma represents the only potential curative option.

## Conclusion

In our single-center analysis, CEA tumor marker levels and tumor grade were identified as significant predictors for poor survival in patients with pancreatic head cancer. However, hard texture of the pancreatic gland appears to exhibit an indirect positive effect on patient survival after pancreatic head resection, but the reason for the effect remains unclear.

## Abbreviations

ICU: Intensive care unit; PDAC: Pancreatic ductal adenocarcinoma; ISGPS: International study group of pancreatic surgery; POPF: Postoperative pancreatic fistula; PPH: Post pancreatic hemorrhage; DGE: Delayed gastric emptying; PD: Pancreatoduodenectomy; MRI: Magnetic resonance imaging; CTx: Chemotherapy; PPPD: Pylorus-preserving pancreatoduodenectomy.

## Competing interests

The authors declare no competing interests. This research received no specific grant from any funding agency in public, commercial, or not-for-profit sectors.

## Authors’ contributions

DM wrote the manuscript, collected the data, interpreted the results and statistically analyzed the data, RF and KS analyzed the data statistically, interpreted the results and critically revised the manuscript, HM collected the data and wrote parts of the manuscript, RF analyzed the data statistically and corrected the manuscript, EF statistically analyzed the data, interpreted the results and critically revised the manuscript, PC gave important manuscript corrections, SHD designed the concept of the manuscript operations and critically revised the manuscript, and GR designed the study, collected data, and drafted the manuscript. All authors read and approved the final manuscript.

## Pre-publication history

The pre-publication history for this paper can be accessed here:

http://www.biomedcentral.com/1471-2482/13/12/prepub

## Supplementary Material

Additional file 1 Patient characteristics and parameters used for statistical analysis. Click here for file
