# Supplementary material for: Evaluation of survival in patients after pancreatic head resection for ductal adenocarcinoma
Source: BMC Surg. 2013 Apr 22;13:12. doi: 10.1186/1471-2482-13-12 (PMC3639824; doi:10.1186/1471-2482-13-12)
Supplement: Additional file 1 — Patient characteristics and parameters used for statistical analysis. [file 1471-2482-13-12-S1.doc]

**Additional file 1: Table S1**

|  |  |
| --- | --- |
| **patients characteristics** | age, sex, abdominal pain, weight loss, obstructive jaundice, diabetes mellitus, nicotine abuse, alcohol abuse, hypertension, obesity, general conditions |
| **preoperative imaging** | pancreatic duct diameter,  common bile duct diameter |
| **intraoperative findings** | portal venous resection, surgeon,  texture of the pancreas soft/hard |
| **postoperative data** | tumor diameter, POPF grade B, POPF grade C, DGE grade B, DGE grade C, anastomotic leakage, wound infection, PPH, cholangitis |
| **tumor characteristics** | tumor grading, pT, pN, pM, R0, Rx, R1, R2, venous infiltration, perineural infiltration, lymphatic infiltration |
| **laboratory data** | Hb, Leukocytes, Thrombocytes, urea, serum proteins, y-GT, ASAT, bilirubin, amylase, lipase, CRP, CEA, CA 19-9 |
|  |  |
